# Proposal and Evaluation of a Physician-Free, Real-Time On-Table Adaptive Radiotherapy (PF-ROAR) Workflow for the MRIdian MR-Guided LINAC

**DOI:** 10.3390/jcm11051189

**Published:** 2022-02-23

**Authors:** Jacob C. Ricci, Justin Rineer, Amish P. Shah, Sanford L. Meeks, Patrick Kelly

**Affiliations:** Department of Radiation Oncology, Orlando Health Cancer Institute, 9900 W Colonial Drive, Ocoee, FL 34761, USA; jacob.ricci1@gmail.com (J.C.R.); amish.shah@orlandohealth.com (A.P.S.); sanford.meeks@orlandohealth.com (S.L.M.); patrick.kelly@orlandohealth.com (P.K.)

**Keywords:** MR-guided radiation therapy, adaptive radiotherapy, contour comparison

## Abstract

With the implementation of MR-LINACs, real-time adaptive radiotherapy has become a possibility within the clinic. However, the process of adapting a patient’s plan is time consuming and often requires input from the entire clinical team, which translates to decreased throughput and limited patient access. In this study, the authors propose and simulate a workflow to address these inefficiencies in staffing and patient throughput. Two physicians, three radiation therapists (RTT), and a research fellow each adapted bladder and bowel contours for 20 fractions from 10 representative patient plans. Contouring ability was compared via calculation of a Dice Similarity Index (DSI). The DSI for bladder and bowel based on each potential physician–therapist pair, as well as an inter-physician comparison, exhibited good overlap amongst all comparisons (*p* = 0.868). Plan quality was compared through calculation of the conformity index (CI), as well as an evaluation of the plan’s dose to a ‘gold standard’ set of structures. Overall, non-physician plans passed 91.2% of the time. Of the eight non-physician plans that failed their clinical evaluation, six also failed their evaluation against the ‘gold standard’. Another two plans that passed their clinical evaluation subsequently failed in their evaluation against the ‘gold standard’. Thus, the PF-ROAR process has a success rate of 97.5%, with 78/80 plans correctly adapted to the gold standard or halted at treatment. These findings suggest that a physician-free workflow can be well tolerated provided RTTs continue to develop knowledge of MR anatomy and careful attention is given to understanding the complexity of the plan prior to treatment.

## 1. Introduction

As technology continues to develop in the field of radiation oncology, the precision of radiation delivery continues to improve [1]. A recent area of rapid improvement is daily adaptive radiation therapy [2]. Specifically, with the MR-LINAC platform, daily adaptive radiotherapy has allowed for dose escalation to targets and greater sparing of surrounding organs at risk (OAR) across multiple sites as compared to traditional image-guided radiation therapy (IGRT) [3,4,5]. However, daily adaptive radiotherapy is available on limited treatment platforms and is labor intensive [6,7,8]. Currently implemented workflows at many institutions require the participation of the entire radiation oncology clinical team: therapists, physicians, physicists, and dosimetrists. This can limit patient throughput and access, particularly for conventionally fractionated daily treatments.

For the historical perspective, radiation therapy has navigated several revolutionary advances to the field which have altered clinical, treatment planning, and treatment delivery workflows. Prior advances include CT simulation, computerized radiation treatment planning and, most relevant to the current discussion, image-guided radiation therapy (IGRT). The integration of IGRT into therapeutic workflows initially involved frequent and direct involvement by multiple members of the clinical team including physicians and medical physicists. This, however, led to delays in treatment delivery and restriction of patient access to this revolutionary new technology. In response, the field adjusted by expanding and redefining the roles of members of the treatment team. This was particularly true for radiation therapists, who are now trained in evaluating volumetric imaging for alignment as opposed to relying only on historical approaches such as portal imaging [9]. The evolving technology also led to the need for a more robust, non-technical quality assurance system to monitor for process errors [10].

Recently, the Orlando Health Cancer Institute (OHCI) has proposed, and evaluated, changes to the real-time adaptive workflow to address staffing inefficiencies and improve patient access and throughput for real-time adaptive treatment on an MR-LINAC. Importantly, our current workflow is defined as adapt-to-shape (ATS), which includes modification of contours during treatment plan adaptation. This is an additional step to an adapt-to-position (ATP) workflow, which only includes transferring contours, but not adjusting those contours. We have pursued a model similar to the precedent set during the adoption of IGRT workflows, by expanding the training and responsibilities of radiation therapists in a physician-free, real-time on-table adaptive radiotherapy (PF-ROAR) workflow for patients receiving daily fractionated radiotherapy. In this study, we describe the development of the PF-ROAR workflow and detail the impacts on staffing requirements and patient throughput. We also performed an analysis of the workflow in a simulated setting to verify the maintenance of plan quality for clinical delivery without direct physician involvement.

## 2. Materials and Methods

To determine the clinical feasibility of a physician-free adaptive workflow, interdisciplinary discussions were held to determine aspects of the current workflow that were problematic. Based upon this review, a novel streamlined workflow was proposed. Through an iterative process, educational needs and potential throughput bottlenecks were identified and addressed. This interdisciplinary team consisted of two radiation oncologists, a physicist, a dosimetrist, and the chief therapist at OHCI. An approved protocol was designed to implement the proposed workflow via an MR-LINAC emulator (ViewRay Inc., Oakwood Village, OH, USA) using previously treated patient plans to assess clinical feasibility and maintenance of plan quality.

### 2.1. Patient Selection and Restoration

Ten representative patient plans that were adapted and treated at OHCI on the ViewRay MRIdian were selected for this study (Table 1). All plans were designed for cancers in the pelvis. Six patients were planned to deliver between 1.8 and 2 Gy per fraction, while four were planned for 5 Gy per fraction. While initial clinical plans may have included targets for a simultaneous integrated boost (SIB), the plans were simplified by removing the high dose target to improve generalizability.

The protocol for MRI-guided radiation therapy to the pelvis for rectal and anal cancer has been standardized in our department. For simulation, patients are instructed to come to the department with a comfortably full bladder and empty rectum. They are taken to the CT simulator suite where an immobilization cast is created to allow for reproducible patient positioning with the MRI coils. Once this is completed, the patient undergoes CT scanning to map the electron density for treatment planning. Next, the patient is taken immediately to the MRIdian system, where a multiplanar MRI scan is performed. The resultant scans are then imported into the ViewRay^®^ treatment planning system and fused.

For treatment planning, the GTV, CTV, and organs at risk (OARs) are contoured by the treating physician on the MRI data set. Note, in the low pelvis, the CTV is contoured to extend 1 cm into the bladder. In the upper pelvis, the CTV is defined by the traditional anatomic landmarks of the vascular system and boney anatomy. Additionally, the CTV is not subtracted out from the bowl. Finally, the following OARs are contoured: bowel (defined as the loops of small and large bowel through the sigmoid colon), bladder, femoral heads, and genitalia. No PRV restrictions are used. Next, using a set of Boolean rules, a planning CTV (CTVplan) is generated automatically by the treatment planning system such that the CTV is trimmed to extend only 3 mm into the bladder and trimmed out of the bowl. Finally, the CTVplan is expanded by 3 mm to generate the PTV. IMRT treatment planning is then carried out in a standard fashion with the goal for a standard course of PTV D95 ≥ 45 Gy, Bladder D_mean_ ≤ 40 Gy, Bowel D(0.03 cc) ≤ 50 Gy, and Femoral head D40 ≤ 37.5 Gy and D_max_ ≤ 44.6 Gy. For a short course delivering 5 Gy per fraction, these are adjusted to PTV D95 ≥ 25 Gy, Bladder D_mean_ ≤ 22 Gy, Bowel D(0.03 cc) ≤ 27.5 Gy, and Femoral head D35 ≤ 20 Gy.

**Figure 1:**
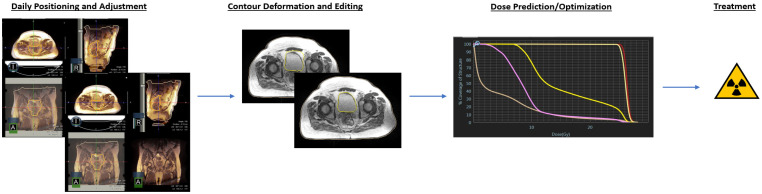
General workflow for ATP adaptive therapy. First, a daily scan is taken and discrepancies between the daily and planning images are corrected. This is represented in the first pair of images on the left. OAR contours are then deformed and corrected. Target contours are rigidly copied to the day’s image. Dose from the plan is predicted given the daily images and reoptimized as needed. Once approved, treatment proceeds.

For treatment, the patient is again instructed to come with a comfortably full bladder and empty rectum. They are set up on the treatment machine, aligned, and shifted based on the boney landmarks of the pelvis. Subsequently, the OARs are re-contoured, and the Boolean rules are re-applied to generate the CTVplan and PTVs for that day’s treatment. Online adaptive treatment planning is then carried out, the plan is approved, and the patient is treated. A general representation is shown in Figure 1.

### 2.2. Participant Selection and Workflow Implementation

To implement the workflow with relevant comparators, six team members were selected for participation: two attending radiation oncologists (one designated as a specialist in pelvic malignancies), three radiation therapists, and a research fellow. The radiation therapists and research fellow received training from the attending radiation oncologists in MRI anatomy, planning system contouring tools, adaptive dose prediction, and adaptive treatment planning. Competency was confirmed by a review of a set of training fractions. Each participant then completed fully optimized adaptive replanning for 2 fractions of each of the 10 patients.

### 2.3. Daily Adaptation Emulation

For each fraction, participants were asked to perform a plan adaptation based on the daily anatomical variations present in each daily positioning scan. The couch shifts were completed by entering pre-determined shift values that were consistent for each participant. Organ structure contours were then deformed to the daily positioning scan. Target contours were rigidly copied from the initial plan to the daily images but were not edited. Participants modified contours for bladder and bowel structures for each fraction. A superior limit 1.5 cm cranial to the CTV was instituted for the bowel. The dose was then predicted using the current image set and optimized for the daily adaptation.

For all plans, participants’ contours were compared by calculating a Dice similarity index (DSI). Contour masks were generated using the Dicompyler and DicomRTTool libraries [11,12] and the DSI was calculated as follows:

$$\mathrm{DSI}=2*\frac{\left(V_{\mathrm{ref}}\cap V_{\mathrm{test}}\right)}{\left(\left|V_{\mathrm{ref}}\right|+\left|V_{\mathrm{test}}\right|\right)}$$

where V_ref_ and V_test_ are the associated volumes of the mask created by the Python script.

Conformity indices for the adapted plans were calculated following the RTOG definition

$$\mathrm{CI}=V_{\mathrm{RI}}/\mathrm{PTV}$$

where V_RI_ is the 95% isodose level.

DSI and conformity indices were evaluated with an ANOVA test where differences were significant if *p* < 0.05.

Plan quality was determined through the use of a standardized set of scaled dose constraints based upon those detailed in NRG-GI002 (Table 2) [13]. Each plan was evaluated by the specialist physician against these constraints for appropriateness for clinical delivery, and a pass rate was determined using an ideal and acceptable variation designation.

Bladder and bowel structures for each fraction that the specialist physician planned were considered as the study’s ‘gold standard’. For each fraction, these structures were exported from the specialist physician’s plan and overlaid onto each non-physician’s associated plan. Finally, the dose to both the non-physician and ‘gold standard’ structures was then evaluated against the constraints described above.

## 3. Results

The interdisciplinary team reviewed the current adaptive workflow to identify the most significant sources of on-table adaptive treatment delivery delay. Over the course of observation, two main issues causing significant treatment delays became apparent.

First, the registration of the daily MR images to the planning MR images can cause delays due to inconsistent breath-hold or bladder-filling. While potentially significant, these delays lie outside of the scope of modified adaptive workflow and will not be addressed further in this work.

Second, the availability of all members of the current adaptive treatment team may lead to delays. The current workflow requires participation by radiation therapists, medical physicists, and a physician. The interdisciplinary team concurred that removal of the physician from the on-table treatment, as occurs with IGRT, would greatly improve patient throughput and access.

**Figure 2:**
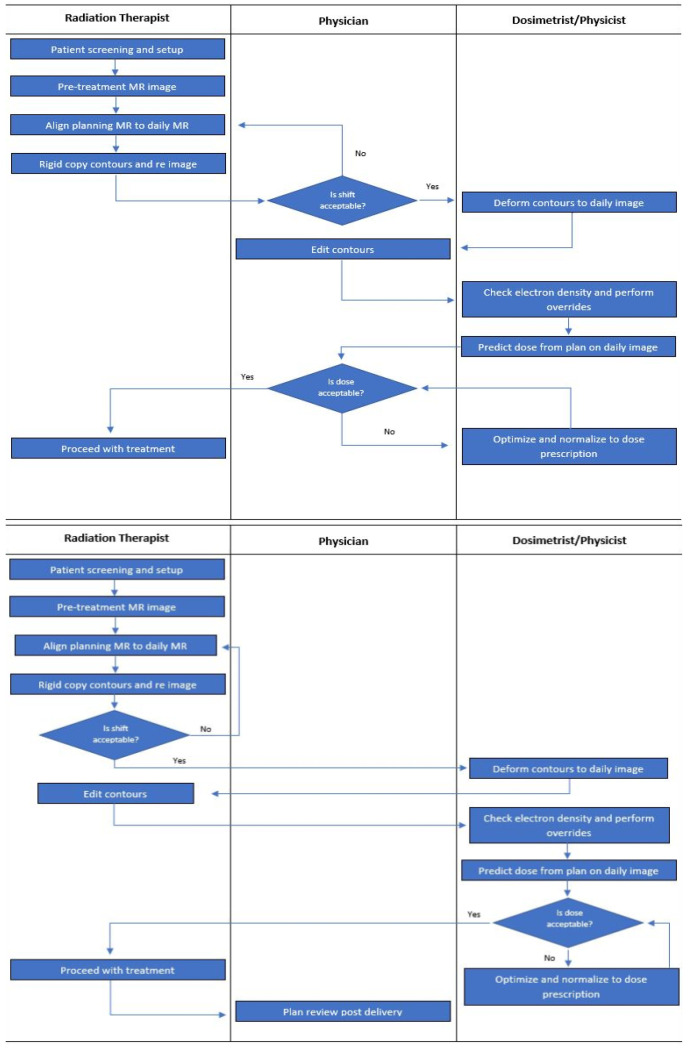
Swim lane depiction of current (top) and proposed (bottom) adaptive radio therapy workflows on an MR-LINAC.

The workflow displaying both the current and proposed distribution of responsibility is shown in Figure 2. The main change to this workflow transfers the responsibility of approving daily couch shifts and editing OAR contours from the physician to the radiation therapist. However, the physician will still conduct an offline review, as a second check, to prevent the propagation of any planning errors that occur in the adaptation process—similar to the workflow with IGRT. Plans that do not meet NRG-GI001 constraints prior to that day’s delivery would trigger an immediate on-table review.

### Plan Evaluations

Table 3 shows the mean and standard deviation values of DSI for bladder and bowel based on each potential physician–therapist pair, as well as an inter-physician comparison. As seen, there is a good overlap amongst all comparisons (*p* = 0.868).

Table 4 shows the resulting average conformity indices for each participant. In all cases, there is good agreement between each participant and the resulting plans achieve acceptable dose conformity.

When evaluated against the institutional planning standard, the physician pass rate was 100%, while the non-physician pass rate was 90%. Among the physician plans, 35/40 plans met protocol constraints and 5/40 were within acceptable variation. For the non-physicians, 68/80 plans met protocol constraints, 4/80 were within acceptable variation, and 8/80 did not meet constraints. These eight fractions would necessitate physician review and possible adjustment prior to treatment.

With respect to the dose to the ‘gold standard’ structures, the non-physicians produced plans that passed 91.2% of the time. Overall, 70/80 plans met protocol constraints, 3/80 were within acceptable variation, and 7/80 did not meet constraints. Of these seven plans, all failed to meet the bowel D(0.03 cc) constraint. The average volume of the ‘gold standard’ bowel at 55 Gy was 0.66 cc (range, 0.06–1.38 cc). Three of these plans had a mean dose to the ‘gold standard’ bladder that was within acceptable variation. All failing plans corresponded to a single patient who was treated for an anal cancer with a large target that included the inguinal lymph nodes.

**Figure 3:**
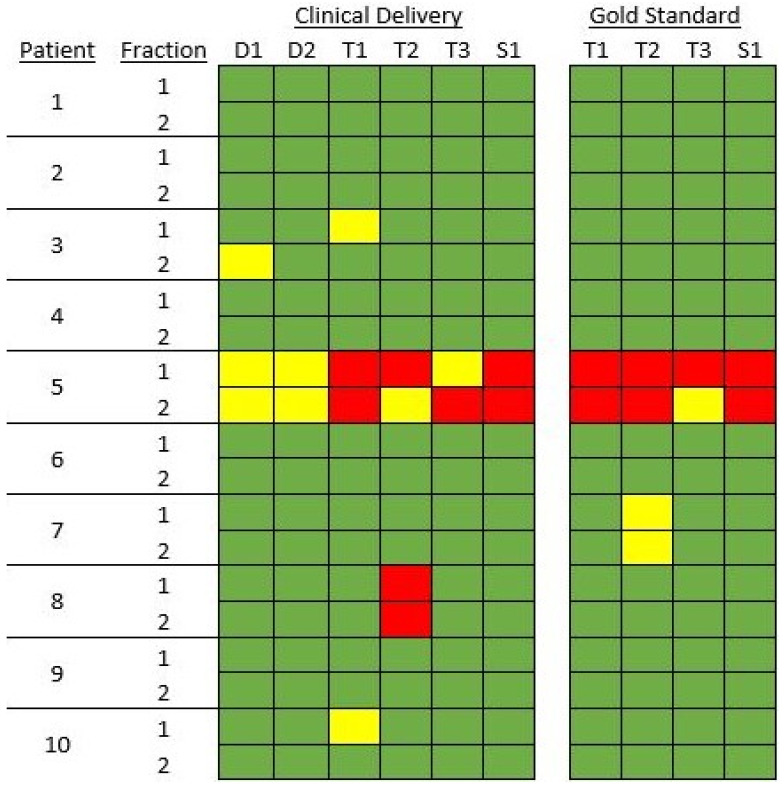
Heat map of plan outcomes. Green boxes signify that the plan met the specified constraints. Yellow boxes signify that the plan was within the ‘Acceptable Variation’ range for one or both constraints. Red boxes signify the plan missed one or both specified constraints.

The distribution of these results is shown in Figure 3. As seen, five of the seven plans that failed the protocol for the ‘gold standard’ structure set also failed evaluation for the clinical delivery. As such, per protocol, these plans would have been flagged for review by the physician prior to treatment. Only two non-physician plans that passed their clinical delivery evaluation failed when their dose was evaluated on the ‘gold standard’. As a result, in 97.5% of cases, the PF-ROAR process would have resulted in an acceptable plan being delivered or prompted an on-table physician review prior to treatment. Only 2.5% of fractions would have been treated after not meeting constraints, but the subsequent offline review process would have prevented these from being propagated to further treatments.

## 4. Discussion

Real-time on-table adaptive radiation therapy represents an enormous shift in the delivery of radiation therapy. Treatment delivered in this manner results in marked improvement in daily treatment plans with early data supporting improved clinical outcomes [6]. With the advent of the new technology, however, comes significant disruption to standard treatment paradigms and workflows with enormous additional time and resource commitments [6,7,8]. To date, the inefficient nature of MRI-guided online adaptive radiation therapy has been limited in our department to cases where the benefits of daily replanning are expected to be high (i.e., stereotactic body radiation therapy, hypofractionated radiation therapy, and reirradiation). The benefits online adaptive therapy can provide for short-course therapy have been well described, particularly for SBRT of the prostate [14,15,16]. This study provides the blueprint for a physician-free, real-time on-table adaptive radiotherapy (PF-ROAR) through the expansion of roles of certain members of the clinical team while maintaining daily adaptive plan quality for carefully selected patients. With the improved efficiency provided by PF-ROAR, we hope to extend adaptive radiation therapy to more standard fractionated courses. In this setting, though daily gains may be smaller, summated over the course of treatment, the value of these daily adaptations could be considerable.

Other authors have begun to explore a similar shift in clinical roles during MR-guided adaptive radiation therapy. Hales et al. have proposed and instituted a ‘clinician-lite’ model for adaptive radiation therapy. In their study, radiation therapists lead the treatment workflow by completing the treatment image registration, treatment plan adaptation using an ‘adapt to position’ methodology, plan evaluation, approval, and treatment delivery without direct physician involvement. Their analysis of 10 prostate cancer patients under treatment revealed physician intervention was required in only 1.5% of fractions. This resulted in a nearly 20% reduction in treatment time for their patients when compared to their standard workflow [17].

In contrast to the work of Hales et al., PF-ROAR aims to progress physician-free radiation therapy from adapt-to-position (ATP) treatment to the more robust adapt-to-shape (ATS) treatment. ATP involves rigid registration between the pre-treatment images and the online daily scan. The dose is then predicted on the images of the day and optimized as needed. In ATP, contours are not adjusted after the registration takes place. Alternatively, ATS offers a much more adaptive plan but also requires a more complex workflow. The pre-treatment images are registered to the daily images as in ATP planning, but the contours are also deformed and adapted to account for the variability in the shape and position of the organs and targets. This has been demonstrated to result in dosimetrically superior adaptive plans, but it is significantly more resource intensive [18].

As PF-ROAR requires more active participation by the radiation therapist, it was not unexpected to see a higher rate of unacceptable plans generated with this approach compared to that of Hales et al. Despite the excellent agreement in all planned fractions for contours tested by the Dice similarity index and plan conformality indices, employing the ‘gold standard’ contour benchmark, only 91.25% of physician-free fractions met constraints or had acceptable variations for clinical treatment. Of the unacceptable fractions for treatment, all occurred in a single patient, patient 5. This plan was the most complicated plan tested as the patient was treated for a T4N0M0 squamous cell carcinoma of the anus with fistulous vaginal involvement. While each fraction planned by the physicians was deemed appropriate for treatment, only one of eight adapted fractions planned without physician input was deemed acceptable for clinical treatment. Excluding this challenging case, 97.2% of non-physician fractions met constraints for clinical delivery. This finding suggests that complex treatment plans may not be appropriate for PF-ROAR. Further, the extension of PF-ROAR to other anatomic sites, simultaneous integrated boost treatment plans, or extreme hypofractionation such as stereotactic body radiation therapy must be carefully evaluated for maintenance of plan quality prior to implementation.

Additionally, this finding highlights the importance of a robust system of checks that would halt the delivery of a fraction that is grossly unacceptable and did not meet the planned constraints. In this study, five of the plans that did not meet constraints on the gold standard contour set also did not meet the clinical constraints at the time of planning. As a result, in these instances, the PF-ROAR process would trigger an immediate on-table review by the treating physician, and the inappropriate plan would not have been treated.

In this study, there were only two fractions that would not have triggered a clinical review but were subsequently found to be unacceptable when evaluated against the ‘gold standard’. In these cases, the offline review would allow the treating physician to determine if there has been a change in the target anatomy that would require physician recontouring of the target and offline replanning. During this step, any errors in alignment, contouring, or planning can be caught, preventing an error on a single fraction from being propagated for the entire treatment.

Changes in clinical care such as those outlined here are dictated in part by the constraints of the environment in which they occur. The PF-ROAR workflow described was developed in the context of the United States healthcare system, accounting for practice guidelines from US credentialing groups, such as those published by the American College of Radiology, regulatory agencies, or any guidelines defined within our payor environment. Outside of this context, the particulars of a physician-free ATS workflow may vary significantly. Full implementation in the US regulatory and payor environment will also require the development of streamlined offline plan review interfaces to allow physicians to thoroughly evaluate delivered plans post-hoc after physician-free adaptive treatment fractions are completed. Furthermore, wider adoption of this change in the clinical role of radiation therapists will require formal changes to their education and training. It will also require a formalized and widely accepted non-technical quality assurance system to monitor for competence and maintenance of plan quality to minimize the impact of operator dependence and process errors.

In conclusion, PF-ROAR represents a streamlined workflow that is efficient and capable of delivering consistent adapt-to-shape radiation treatment plans which are clinically acceptable and of high quality in carefully selected patients. Additional evaluation is needed to determine this workflow’s appropriateness in other treatment settings and regulatory environments.

## Figures and Tables

**Table 1:** Diagnoses, plan dose, and total fractions for the patients chosen for this study. An asterisk signifies the dose includes an SIB target which was subsequently removed for the purposes of this study.

Patient	Diagnosis	Dose (Gy)	Fractions
1	Rectal Adenocarcinoma with lymph node involvement	52.5 *	25
2	Rectal Adenocarcinoma	25	5
3	Moderately differentiated adenocarcinoma of the sigmoid	63 *	28
4	Rectal Adenocarcinoma	25	5
5	Squamous cell carcinoma of the anus with vaginal fistula	58 *	29
6	Rectal Adenocarcinoma	45	25
7	Rectal Adenocarcinoma	45	25
8	Ovarian cancer with lymph node metastases	15	3
9	Adenocarcinoma of the colon	25	5
10	Rectal Adenocarcinoma	45	25

**Table 2:** OAR dose constraints for plan evaluation based on dose per fraction.

Dose/fx (Gy)	Bladder D_mean_	Acceptable Variation	Bowel D(0.03 cc)	Acceptable Variation
1.8–2	≤40 Gy	≤44	≤50 Gy	≤55 Gy
5	≤22 Gy	≤24	≤27.5 Gy	≤30 Gy

**Table 3:** Mean and standard deviation values for Dice similarity indices (DSI) for bladder and bowel structures for each pair-wise comparison of physician, therapist, and student. The comparison between physicians served as a benchmark to judge resulting outcomes.

	D1 v D2		D1 v T1		D1 v T2		D1 v T3		D1 v S1	
	M	SD	M	SD	M	SD	M	SD	M	SD
Bladder	0.98025	0.029833	0.96905	0.061505	0.97605	0.035744	0.978795	0.036447	0.965915	0.067395
Bowel	0.98965	0.022475	0.99185	0.020575	0.97305	0.062442	0.96571	0.121103	0.9842	0.028666

**Table 4:** Average and standard deviation values for conformity indices across all plans and participants. Conformity indices were calculated according to RTOG protocol using the 95% isodose line as the reference isodose.

	D1		D2		T1		T2		T3		S1	
	M	SD	M	SD	M	SD	M	SD	M	SD	M	SD
CI	1.119	0.272	1.145	0.093	1.209	0.184	1.251	0.252	1.149	0.074	1.144	0.080

## Data Availability

Data will be made available upon request to the corresponding author.
